# 875 Effect of Continuous Albumin Use on Fluid Resuscitation Volume in Burn Shock Patients

**DOI:** 10.1093/jbcr/iraf019.406

**Published:** 2025-04-01

**Authors:** Luke Posgai, Beatrice Adams, Nicolas Tran, Ram Velamuri

**Affiliations:** Tampa General Hospital Regional Burn Center; Tampa General Hospital; Tampa General Hospital; University of South Florida Morsani College of Medicine

## Abstract

**Introduction:**

Fluid resuscitation is an essential component of burn care, yet optimal resuscitation techniques have not been well defined. Colloids are often added to standard crystalloid resuscitation in an effort to reduce fluid volume demands and subsequent complications of over resuscitation.

**Methods:**

We conducted a single center, retrospective, chart review that included adults with 40% or greater TBSA burn who presented before and after the development of a colloid resuscitation protocol. During both the pre- and post-protocol periods, initial crystalloid fluid estimates within the institution were 2 mL/kg/%TBSA for general burns (age >= 13 years), 3 mL/kg/%TBSA for pediatric burns (age <= 12 years), and 4 mL/kg/%TBSA for electrical burns (high voltage with evidence of myoglobinuria). Subsequent fluid titration as fluid boluses were left to the discretion of the critical care provider in patients not meeting urine output or hemodynamic goals. The colloid resuscitation protocol that included a 5% albumin continuous infusion at a dose of 0.5 mL/kg/%TBSA over 16 hours be considered if crystalloid based volume resuscitation failed to maintain hemodynamics or urine output goals after 24 hours.

**Results:**

A total of 28 patients were included, 15 patients and 13 patients from the pre- and post-protocol periods, respectively. Use of continuous albumin was associated with a significant reduction in total fluid volume administration at 48 hours with a median volume of 7.56 mL/kg/%TBSA in the pre-protocol group compared to 5.08 ml/kg/%TBSA in the post-protocol group, P = 0.04. The percentage of patients with a 24-hour Ivy index exceeding 250 mL/kg was not statistically different but was numerically lower in post-protocol patients, occurring in 66.7% of pre-protocol patients and 38.5% of post-protocol patients, P = 0.25. There was no statistically significant difference between pre- and post-protocol groups in the attainment of urine output or mean arterial pressure goals during any of the time periods. However, a greater proportion of patients in the post protocol group received RRT (26.7% vs 69.2%, P = 0.06).

**Conclusions:**

This study found that the use of continuous albumin was associated with a significant reduction in total fluid administration during the acute resuscitation of burn shock in patients with > 40% TBSA burns.

**Applicability of Research to Practice:**

Although early crystalloid resuscitation is a foundational component of burn care, more recent evidence has highlighted the importance of avoiding over resuscitation and its associated sequelae. Albumin has historically been utilized for this purpose; however, controversy remains as to the optimal strategy for albumin use in fluid resuscitation, particularly as it relates to the timing of initiation.

**Funding for the Study:**

N/A

